# Enabling Remote Health-Caring Utilizing IoT Concept over LTE-Femtocell Networks

**DOI:** 10.1371/journal.pone.0155077

**Published:** 2016-05-06

**Authors:** M. N. Hindia, T. A. Rahman, H. Ojukwu, E. B. Hanafi, A. Fattouh

**Affiliations:** 1 Wireless Communication Centre, Universiti Teknologi Malaysia, Skudai 81310, Malaysia; 2 Department of Electrical Engineering, Faculty of Engineering, University of Malaya, Kuala Lumpur, Malaysia; 3 Department of Computer Sciences, Faculty of Computing and Information Technology, King Abdulaziz University, Jeddah, Saudi Arabia; Nankai University, CHINA

## Abstract

As the enterprise of the “Internet of Things” is rapidly gaining widespread acceptance, sensors are being deployed in an unrestrained manner around the world to make efficient use of this new technological evolution. A recent survey has shown that sensor deployments over the past decade have increased significantly and has predicted an upsurge in the future growth rate. In health-care services, for instance, sensors are used as a key technology to enable Internet of Things oriented health-care monitoring systems. In this paper, we have proposed a two-stage fundamental approach to facilitate the implementation of such a system. In the first stage, sensors promptly gather together the particle measurements of an android application. Then, in the second stage, the collected data are sent over a Femto-LTE network following a new scheduling technique. The proposed scheduling strategy is used to send the data according to the application’s priority. The efficiency of the proposed technique is demonstrated by comparing it with that of well-known algorithms, namely, proportional fairness and exponential proportional fairness.

## Introduction

In today’s world of high demanding and fast evolving lifestyles, elder citizens are facing many challenges especially related to health issues. Recently, the world’s population has reached a staggering milestone of 7 billion and continues to grow with geometric progression, resulting in increased pressure on medical care and health systems all across the globe [1]. In most developing countries, for example, the issue of poor healthcare infrastructures has had a disproportionate effect on the wellbeing of their growing populations due to the insufficient number of health care units and shortage of doctors to cope with the demand of the increasing population. The main cost of treating diseases in a developing nation lies not so much in the cost of the infrastructure or medication, but rather in the cost of not having enough basic diagnostic equipment for timely diagnosis of illnesses, including life threatening diseases. As a possible solution to solve this problem, developing countries are planning to build more hospitals, train an adequate number of doctors, and equip hospitals with state of the art diagnostic instruments; however, this approach is a very resource intensive and time consuming process.

The best approach that suits the narrated scenario is to propose a system that is capable of remote monitoring the vital signs of patients around the clock, such as heart rate, body temperature, glucose level, blood pressure, etc., and that is capable of communicating with the cloud and automatically placing requests for assistance whenever necessary [2–4]. Such a system is referred to as a “mobile health monitoring system” and constitutes largely of several wearable sensors [5]. With such a system in place, a wider population can be easily reached and provided with on demand medical assistance. Medical services can be provided by a designated specialist doctor among a group of specialized doctors from any part of the world; he or she is able to evaluate the parameters on the cloud [6]. Subsequently, if a health sensing device is configured to communicate with a portable computing device such as a tablet or a smartphone, which by default is capable of communicating with the cloud, then the whole system can be made much more cost effective. Currently, most people have access to these portable communication devices, and these devices have become quite affordable.

In this paper, we have proposed a new paradigm shift in health-care monitoring systems based on two stages. In the first stage, sensors transfer collected data to an android application. In the second stage, a new scheduling technique is proposed to send the patient’s data that depends on the sensitivity of the patient’s condition. Based on the authors’ knowledge, this is the first time in which such a system has been proposed.

The rest of this paper is organized as follows. Section 2 describes the related study of the Internet of Things, wireless sensor networks, and scheduling over LTE-Femto cell networks. Section 3 discusses the system model, and Section 4 clarifies the simulation environment. The results and discussion are illustrated in Section 5. The limitation of this study and future works are discussed in Section 6. The conclusion is presented in Section 7.

## Related Study

### Internet of Things

The last couple of years have experienced a significant growing interest in the technology of the Internet of Things (IoT). Such a growing interest has been motivated by the capabilities and opportunities that the IoT will offer when fully deployed [7–9]. The IoT, among other things, has promised to create a world of interconnected objects, i.e., every object within and outside of our reach will be interconnected and will communicate with each other over the internet with little or no human intervention. This will invariably create an intelligent world where objects act smart enough to respond to the immediate needs of a person without explicit instructions. The interconnection of our everyday objects means that many applications will be enabled in various domains including the application domain, which can be further subdivided into three main categories based on the following focuses: industry, environment, and society.

The IoT is defined as things having identities and virtual personalities operating in intelligent spaces and using smart interfaces to connect and communicate within social, environmental, and sensor scenarios [10–11]. It can be considered as the next generation of the internet that connects everything to other things. In this context, every object is given a unique identifier in the network. This allows the remote access of devices through the network at any time and from any location. The IoT makes it possible for objects to interact with sensors, to access information over the Internet, and to communicate with one another through information sharing, thereby creating intelligent, ubiquitous, and ever connected environments. The IoT also enables Machine to Machine (M2M) communication, which enables machines to autonomously control other machines over the internet [12]. This evolution has the potential to transform the way technology is used because machines will take charge of other machines, thus circumventing the constraints that people encounter while operating electronic systems. For example, machines will monitor sensors globally to generate and analyze terabytes of valuable data in a matter of seconds that otherwise would take years of human effort to achieve. The IoT makes the concept of pervasive and ubiquitous computing a reality by making it possible for the objects of our daily lives, such as cars, roadways, wrist watches, typical household electronics appliances, including refrigerators, TVs, fans, and air-conditions, pill-shaped cameras in our digestive tubes, and even domestic or wild animals for taming purposes, to be equipped with sensors for tracking [10].

### The Essential Component of the IoT: Wireless Sensor Networks

A Wireless Sensor Network (WSN) is a network of clustered sensors interconnected by means of different technologies and protocols [13, 14]. Sensor networks are normally implemented in one of the three following unique architectures: a **flat architecture,** in which data are transferred from static sensor nodes to an aggregate node using a multi-hop mode; a **two-layer architecture,** in which more static and mobile aggregate nodes are deployed to collect data from sensor nodes; and a **three-layer architecture** (adopted in IoT technology), in which multiple layers of sensor networks are interconnected via the internet.

Due to the traveler’s predicament, the conducts of human movement can be modeled using a game, and recent evolution in game theory has offered a platform to investigate the various dilemmas in social settings where the instrumentality of the IoT can be applied as shown in Ref. [15], in which a modified spatial traveler’s dilemma game based on a two-coupled lattice was proposed by the authors. In this approach, the corresponding players (i.e., patients) on two lattices are integrated into the scheme that attempts to emulate a target player, and the coupling effect of the integration between the corresponding players and the emulation probability are multiplied by a coupling factor, which describes the behavior of a portion of the players on the other network that fall within the estimated interval for this target player, based on the classical Fermi rule. Furthermore, the authors of Ref. [16] proposed strategies to predict the movement of a mobile entity at certain areas and to investigate the mobility and level of cooperation that exists among mobile entities. The proposed model determines the level of cooperation among mobile entities placed on a sparse-square network and autonomously estimates their current conditions, which are then studied using different models based on several mobility strategies and rules to correctly model real situations. An investigation on the weak prisoner’s dilemma on free-scale and random networks was carried out in Ref. [17]. In this study, the game was technically played by active agents with their neighbors and payoffs were awarded based on their participation; inactive players obtained null payoffs because they did not play. Therefore, only the active players successfully relayed their strategies to their neighbors, whereas the inactive players could not replicate these strategies. However, the simplest strategies exhibit incredible complexity in evolutionary dynamics, involving cascading failures that manifest as asymmetric dynamic instabilities in the time course of the evolution of the two competing strategies.

Wireless sensors are the most deployed types of sensors in recent times [18]. Over the course of time, various wireless technologies have been used to establish wireless sensor networks, including a Wireless Personal Area Network (WPAN) (e.g., Bluetooth), Wireless Local Area Network (WLAN) (e.g., Wi-Fi), and Wireless Metropolitan Area Network (WMAN) (e.g., WiMAX, LTE) [16–22].

### Layers in Sensor Networks

As a common procedure, the data generated from the sensor nodes are sent to mobile nodes from all sensors. Then, the mobile node sends the data to the base station, which in turn relays the data to the main controller in the cloud for further processing. Finally, the data reaches the cloud, where the data are supposed to be saved and processed (e.g., national hospital cloud). We have identified the major characteristics in the IoT application of sensor networks, which are as follows [23].

### Intelligence

This means knowledge acquisition. This initial stage involves generating the necessary knowledge by collecting the data and reasoning the data. Converting the acquired raw data into high-level information (knowledge) can be accomplished by collecting a series of data, reasoning the context of the data, and then modeling the scenarios. Based on the context, sensor data can be merged together to deduce new knowledge. The moment that knowledge is obtained, it can be used for more intelligent interaction and communication.

### Architecture

The IoT should be supported by a hybrid architecture that contains many different architectures. Primarily, an **event driven** and **time driven** integrated architecture will be the ultimate configuration for the IoT. In this setup, some sensors (e.g., heart sensors) will automatically generate and transmit data only when certain events occur, while the rest would generate data constantly based on predefined time periods (e.g., temperature sensors) [24]. The IoT and Sensor Networks are mostly event driven [25]. Such systems are commonly built on the principle of Event-Condition-Action (ECA) rules. It is not feasible to connect sensors manually to middleware or applications. Rather, this can be achieved through automated or semi-automated processes. To accomplish this task, applications should exhibit a certain level of understanding and compatibility with the sensors (e.g., data structures they produce, sensors’ capabilities, and driver level/hardware configuration details).

### LTE-Femto Cell Networks

LTE-Femto cells offer low-cost solutions for indoor household and enterprise applications. A single household Femto cell can provide coverage for as few as ~ 4 authenticated sensors confined within a radius of 10–25 meters. A Femto-cell is typically characterized by its low power, short range, and low cost as well as by its aptitude to provide ubiquitous access to outdoor macro cell networks via a broadband backhaul connection, such as a digital subscriber line, optical fiber, or cable modem. The deployment of LTE-Femto cell networks can offer several potential benefits for both mobile operators and consumers [26]. Primarily, they can guarantee better coverage to areas currently not satisfactorily covered with service provided by a Macro-cell. Moreover, the average cost for each megabyte of traffic operated within a Femto cell coverage area is estimated to be cheaper than that of a Macro-cell. Thus, sensors can take advantage of the reduction of costs related to their generated traffic [27–29].

Several scheduling algorithms that have been applied for LTE Macro-cell networks can be applied for LTE-Femto-cell networks as well. These include well-known scheduling strategies, such as Proportional Fairness (PF), Exponential Proportional Fairness (EXP/PF), and Exponential Rule (EXP-Rule) strategies.

In Refs. [30, 31], PF is shown to provide services for non-real time sensors. The scheduling metric is calculated based on prioritizing a sensor with a maximum ratio of the instantaneous to the average data rate of previous transmissions until the current TTI. PF does not account for the delay metric; thus, it is not appropriate for serving real time applications.

There are several algorithms that have been proposed to extend the performance of PF, such as EXP/PF [32]. EXP/PF assigns priorities to sensors based on the nature of their packets. For instance, for real time sensors, EXP/PF is based on an exponential function that guarantees the delivery of real time applications within the delay boundary and, at the same time, maximizes the system throughput. In contrast, for non-real time sensors, PF strategies are used, but these strategies use a weighted factor to determine the number of real time sensors. The main drawback of this algorithm is the positive probability of the drop of services from non-real time applications, which is slightly extended to real time ones.

The EXP-Rule algorithm [33] can be considered as a further enhancement of the EXP/PF algorithm with a significant focus on servicing real time applications, which can be detrimental to non-real time applications. Although these scheduling algorithms exhibit high performance in practical scenarios, they are not flexible enough to adapt to changes of the sensor status and application demands.

### Proposed model

To enable the data transmission of health-care systems, there are two main prerequisites. The first one is the android application that collects data from bio-sensors, and the second one is the mobile station that sends these data over LTE-Femto Cell networks to health-care centers for further analysis (S1 Text. Data Collection and Android Applications).

### Mobile Application Module (MAM)

**Fig 1:**
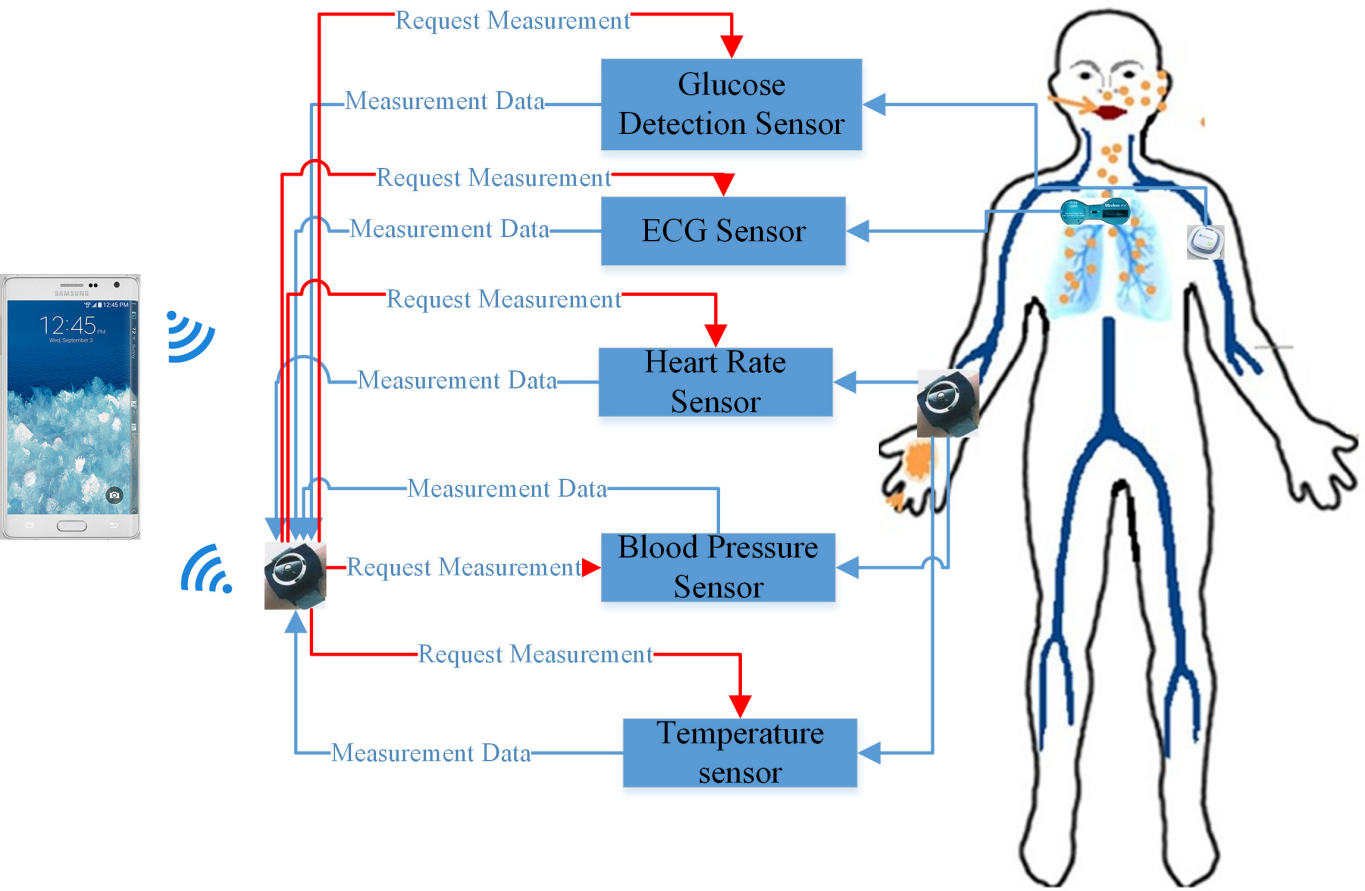
Sensors of the overall human body.

We assume that bio-data, such as physical or physiological health parameters, are gathered from the overall network of sensors attached to the patient. These sensors include a heart rate monitoring sensor, temperature sensor, blood pressure sensor, glucose sensor, and ECG sensor (Fig 1). All of these sensors periodically synchronize with the mobile node, which sends data sequentially and securely to the femtocell and subsequently to the cloud. This sequence is directly related to the patient’s situation and is predefined by the medical consultant section. Any mobile device equipped with the android operating system and Bluetooth facility can be used to accomplish these tasks. Customizable application software can then be developed for the medic to specify event thresholds for patients in critical cases, which triggers an alarm when the threshold limit is reached (S1 Fig).

#### First Phase: Android application (Data collection phase)

**Fig 2:**
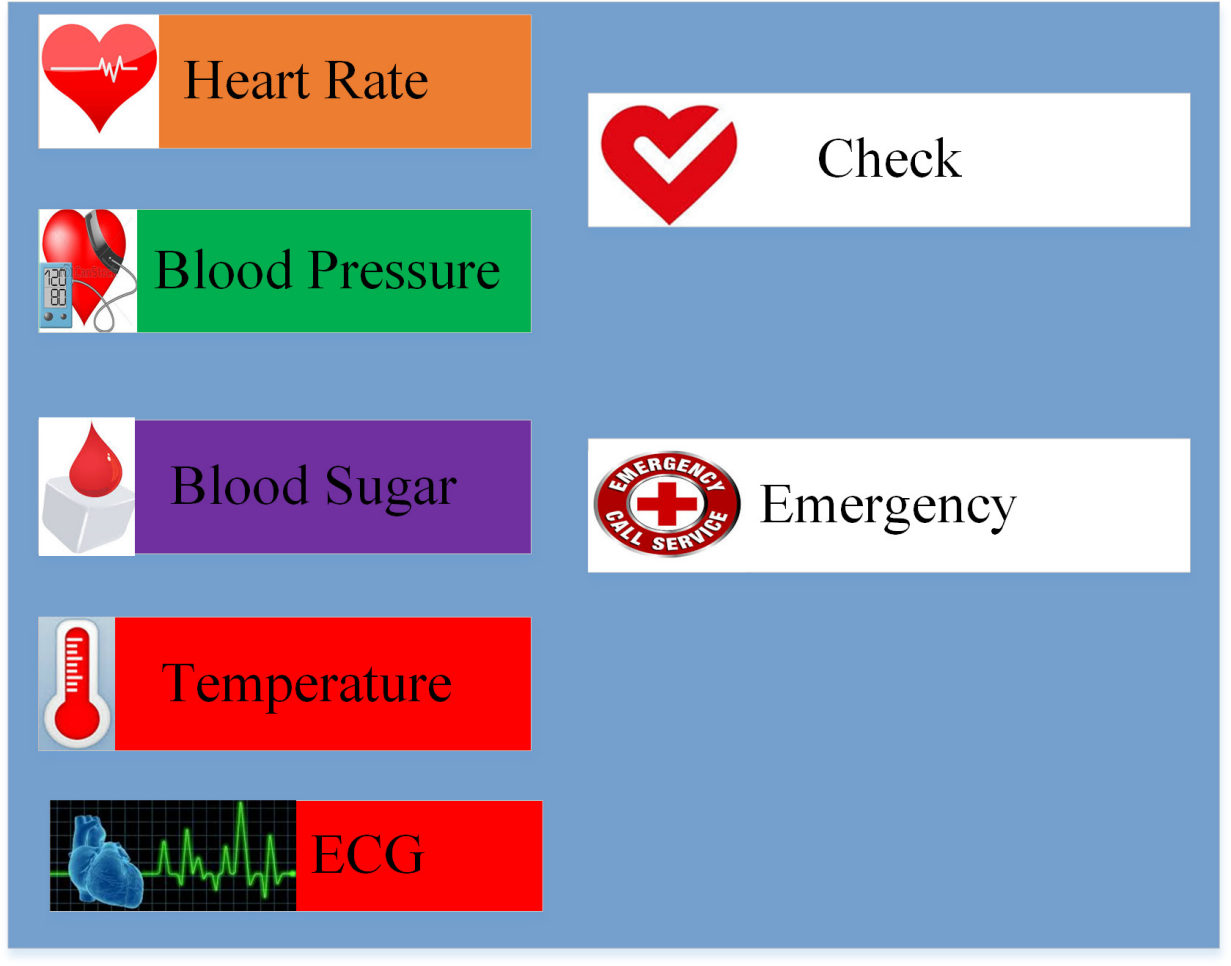
The main android program interface.

Initially, the android application synchronizes information with the sensors for updating the patient’s situation (Fig 2). The collected information is evaluated to determine the current state of the patient’s health condition. If the patient is in critical condition, the event threshold agent triggers an emergency signal, and the android application will automatically send a request to emergency services and/or the ambulance.

For simplicity, we assume that by default all the bio-data are collected from the sensors and are saved in the mobile device’s database. The sensors are explained as follows:

1. ECG Sensor: An electrocardiogram is used to observe the electrical and muscular activities of the heart.
2. Temperature, Blood Pressure, and Heart Rate Sensor: This sensor is a multi-functional sensor and is placed inside a smart watch in physical contact with the body for data collection. It measures the body temperature, blood pressure, and heart rate.
3. Glucose Detection Sensor: This sensor measures the approximate concentration of glucose in the blood.

#### Second Phase: Data Transmission over a Femto-LTE Network

We assume that the interfacing mobile application gathers data from the sensors and transmits them to a Femto-cell Access Point (Femto-cell AP), which is installed at multi-locations around the entire hospital. A patient can move about within the area under the coverage of a Femto-cell, while his sensors periodically synchronize with the mobile application that stores the data at the Femto-cell AP that covers the particular area. The data are then scheduled for onward transmission by using the proposed scheduling strategy (S1 Text. Data Collection and Android Applications).

**Fig 3:**
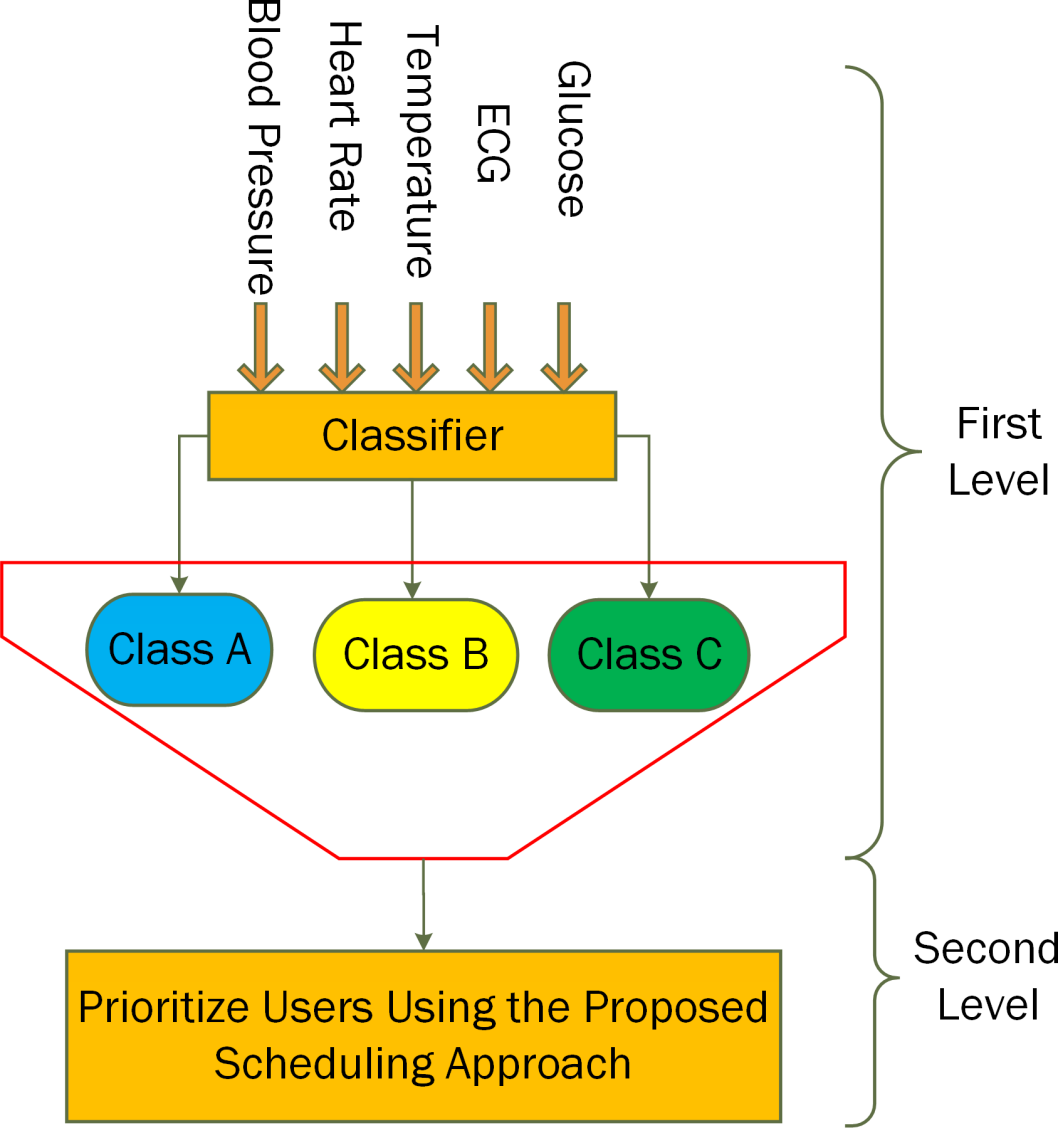
Main system diagram.

As shown in Fig 3, at the first level, we have classified the patient data into three main categories, which include Critical (Class A; combines the data of three main sensors namely **Blood Pressure, Temperature, and Heart Rate** sensors), Chronic (Class B; combines the data of the **ECG** sensor), and Normal (Class C; combines the data of the **Glucose** sensor). On the second level, the studied scheduling approach is used to schedule the data between the Femto-cell and LTE base station. The scheduling decision is based on three main features described below (S1 Text. The Proposed Algorithm)

1. The ratio of the spontaneous data rate at time *t* (*r*_*i*_(*t*)) of sensor *i* to its past average throughput until the present TTI $\bar{R_{i}}\left(t\right)$ (1).
2. The weighting factor (*w*_*i*_) for sensor *i* is based on the patient’s status; for instance, if the patient is hypertensive, the highest priority will be given to Class A, then Class B, and finally Class C data. The weighting factor is directly related to the importance of the practical IoT application *a* (*w*_*IoT*,*a*_*)* and the delay of the sensor’s packets at the queue with respect to the maximum delay budget of practical Class (2). The delay weight factor of sensor *i* (*w*_*di*_) is calculated as the ratio between the head of the line packet delay of sensor *i* and the delay threshold of practical Class (3). It is worth mentioning that the set of weight among the three classes is 1 (4).
3. The packet loss weighting metric of sensor *i* (*α*_*i*_), which is subjected to the acceptable loss rate of sensor *i* (*λ*_*i*_) and the maximum delay budget of sensor *i* (*τ*_*i*_) (5).

$$\begin{array}{l} k_{i}=\arg\;\max\;w_{i}\left(\alpha_{i}\times \frac{r_{i}\left(t\right)}{\bar{R_{i}}\left(t\right)}\right)\\ where\;\bar{R_{i}}\left(t\right)=\beta \bar{R_{i}}\left(t-1\right)=\left(1-\beta )r_{i}\left(t\right)\right) \end{array}$$ (1)

$$w_{i}=w_{\left(IoT,a\right)}+w_{di}$$ (2)

$$w_{di}=\frac{HOL_{i}}{\tau_{i}}$$ (3)

$$\sum_{a=1}^{3}w_{\left(IoT,a\right)}=1$$ (4)

$$\alpha_{i}=\frac{\log\lambda_{i}}{\tau_{i}}$$ (5)

where *β* is a constant related to the window size *T*_*f*_, *HOL*_*i*_ (*t*) is the head of line packet delay of sensor *i*, and *τ*_*i*_ is the maximum delay budget of sensor *i*.

## Simulation Area

The simulation was conducted using LTE-Simulink built on the C++ platform. One cell is considered in this scenario, which includes noises and interferences. The propagation model, such as the simple path loss, is based on the distance from the base station in which multipath losses are considered. Furthermore, shadowing based on a log normal distribution (0 dB and 8 dB) and a penetration loss with 12 dB is used. The fairness among each sensor is measured using the Jain’s fairness method [34]. The IoT application is illustrated in Table 1.

**Table 1:** Application demands of the IoT.

IoT Application	Bandwidth Requirement [kbps]	Delay Budget [ms]
**Class A**	242	100
**Class B**	12	300
**Class C**	0.11	2000

## Results and Discussion

As mentioned earlier, patient data have been categorized into three distinct classes, i.e., A, B, and C, and prioritized based on the patient’s medical condition. For instance, for a patient with a hypertension condition, which is considered as a critical case (Class A), the most important information will include the blood pressure, heart rate, ECG, and glucose.

**Fig 4:**
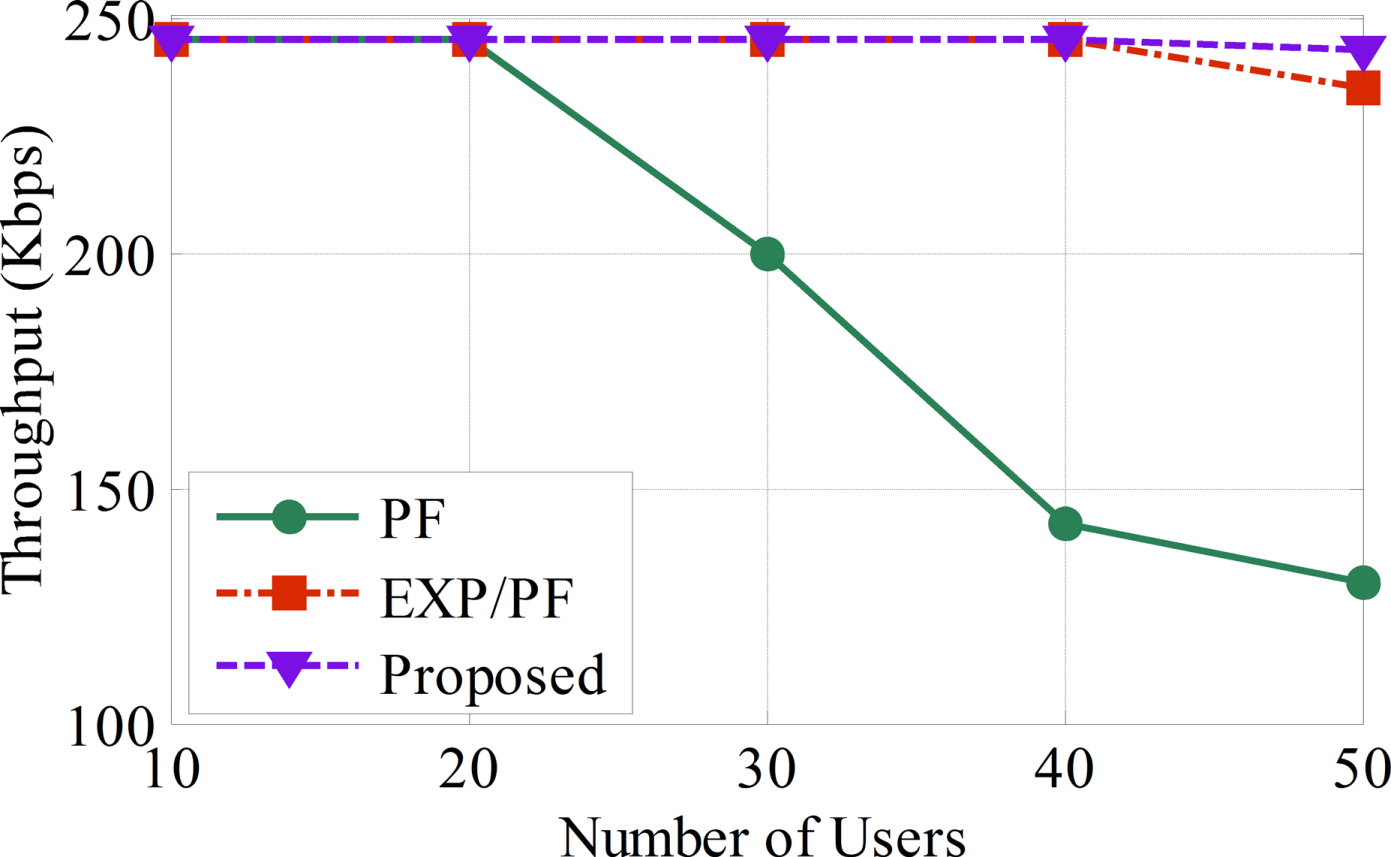
The average throughput per sensor for blood pressure, temperature, and heart rate applications (Class A).

The proposed algorithm exhibits clear evidence of superior throughput performance compared with its counterparts, such as the PF and EXP/PF, as illustrated in Fig 4. The proposed scheme is shown to be at its best level of performance when serving up to 45 sensors at a time; it then begins to dwindle gradually as more sensors are added. The reason behind the robustness of the proposed algorithm is because of the dynamic scheduling approach it adopts. The scheduling decision is directly dependent on the weighting factor. The sensor with the highest weighting factor has the highest priority. For instance, Class A sensors will have the highest weighting factor compared with sensors belonging to other classes. This is in contrast to the PF algorithm, which fails to provide service for Class A sensors because it is designed to serve sensors with the best channel conditions first regardless of the delay factor, i.e., the case with the applications of real time sensors. Hence, PF is considered to be an unreliable approach for providing support for Class A applications. The EXP/PF algorithm shows a performance close to that of the proposed approach and obviously performs better than the PF algorithm for Class A applications. The reason behind this is the fact that the EXP/PF algorithm prioritizes users by exploiting the advantages of the exponential function to guarantee the delay boundaries of real time applications as well as to maximize the system throughput. However, the main issue with such a scheduling approach is its inability to function efficiently, especially in the event that the patient’s dynamic preferences are changed. For instance, if the doctor requests a patient’s glucose level on the spot, it will not be able to meet the change in demands because the EXP/PF approach only works best with a static monitor. With these drawbacks in mind, our proposed model is carefully designed to overcome the shortcomings in both PF and EXP/PF strategies (S2 Fig).

**Fig 5:**
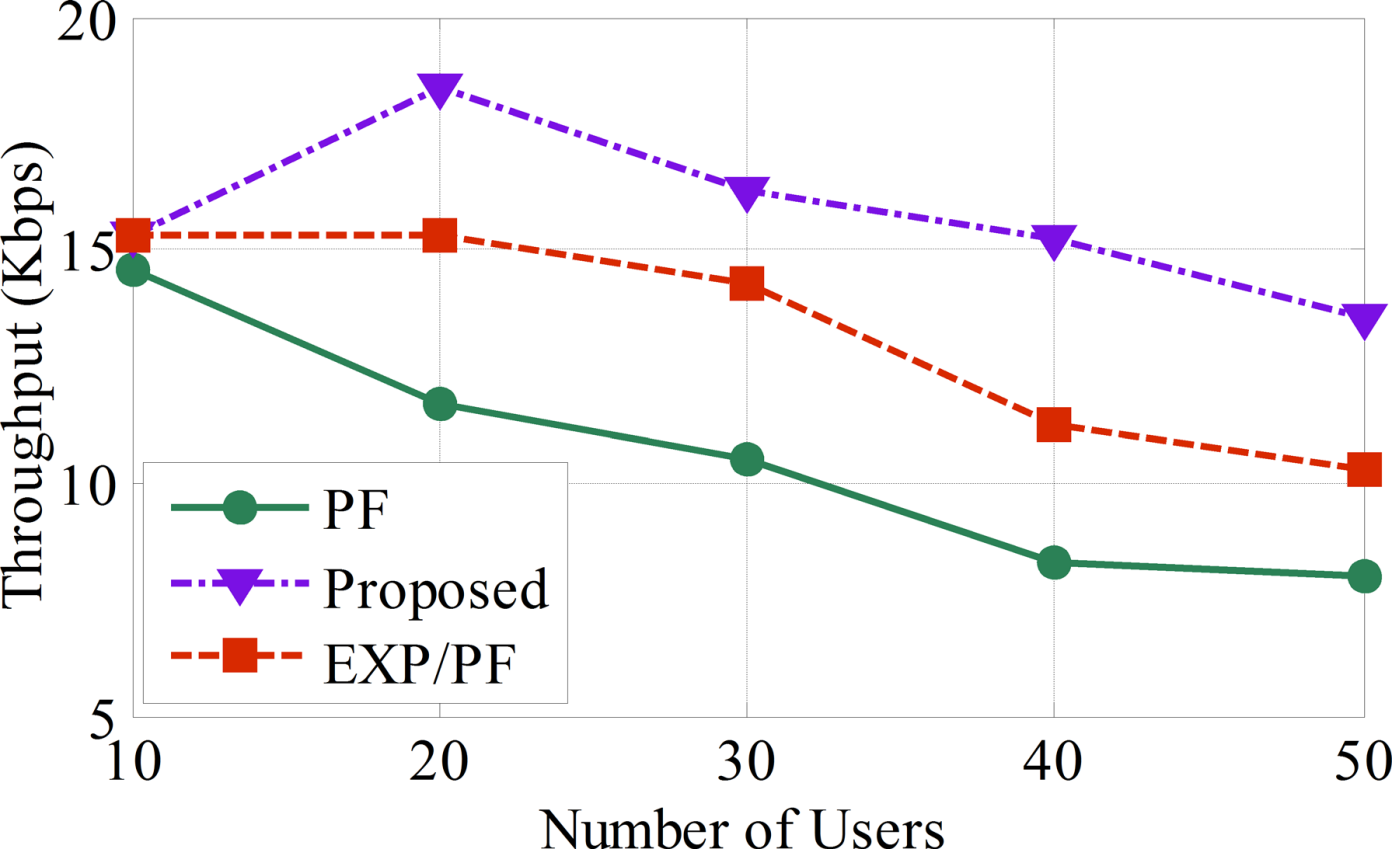
The average throughput per sensor for ECG application (Class B).

From Fig 5, we can conclude that the proposed approach shows the highest performance by sufficiently serving up to 50 sensors compared with the EXP/PF and PF approaches. This is because the proposed approach considers the packet loss factors in its scheduling decision and is able to change dynamically to keep the packet drop rate at a lower level. Meanwhile, the EXP/PF approach allocates less resources to the ECG sensors because there are extra resources that have been allocated to Class A’s sensors. In fact, the EXP/PF approach has no balancing in terms of the resource allocation because the highest priority is given to real time applications (Class A sensors) at the expense of other sensors’ applications, which is detrimental to non-real time applications.

**Fig 6:**
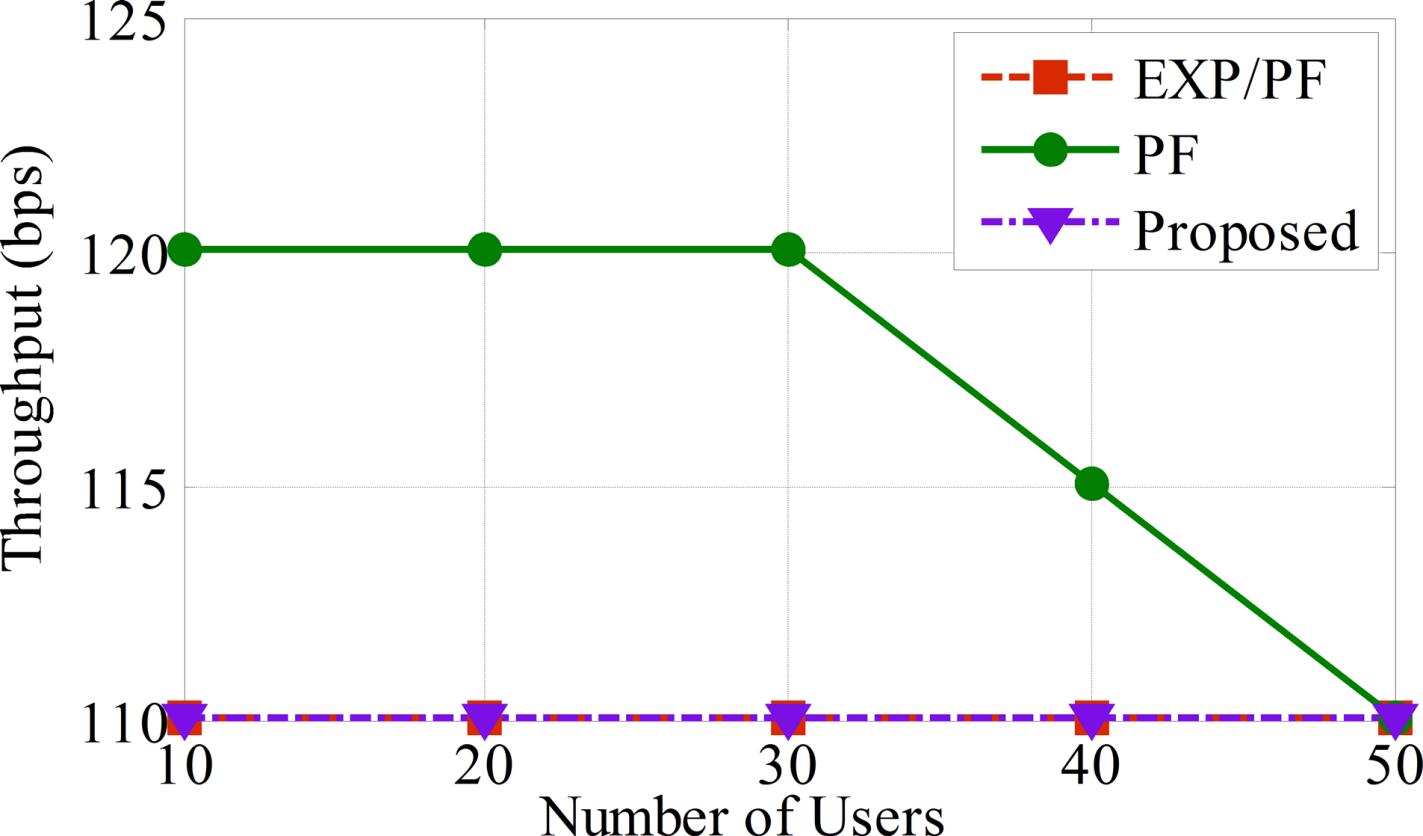
The average throughput per sensor for glucose applications (Class C).

The PF approach shows the lowest performance compared with the other approaches, and this sharp drop of performance is simply because the PF approach is designed to provide a fairness throughput ratio among the sensors regardless of the delay metric. As a result, less resources are assigned to Class A and Class B applications compared with Class C applications, as shown in Figs 4, 5 and 6, respectively (S3 Fig).

The three scheduling approaches for the glucose application are shown below in Fig 6, which shows nearly the same performance. The explanation for this phenomenon is that the glucose application requires the lowest data rate (a few hundred kbps) and, because the LTE network has the ability to provide up to 100 Mbps, all of the scheduling approaches can provide the demanded service for the glucose application efficiently.

**Fig 7:**
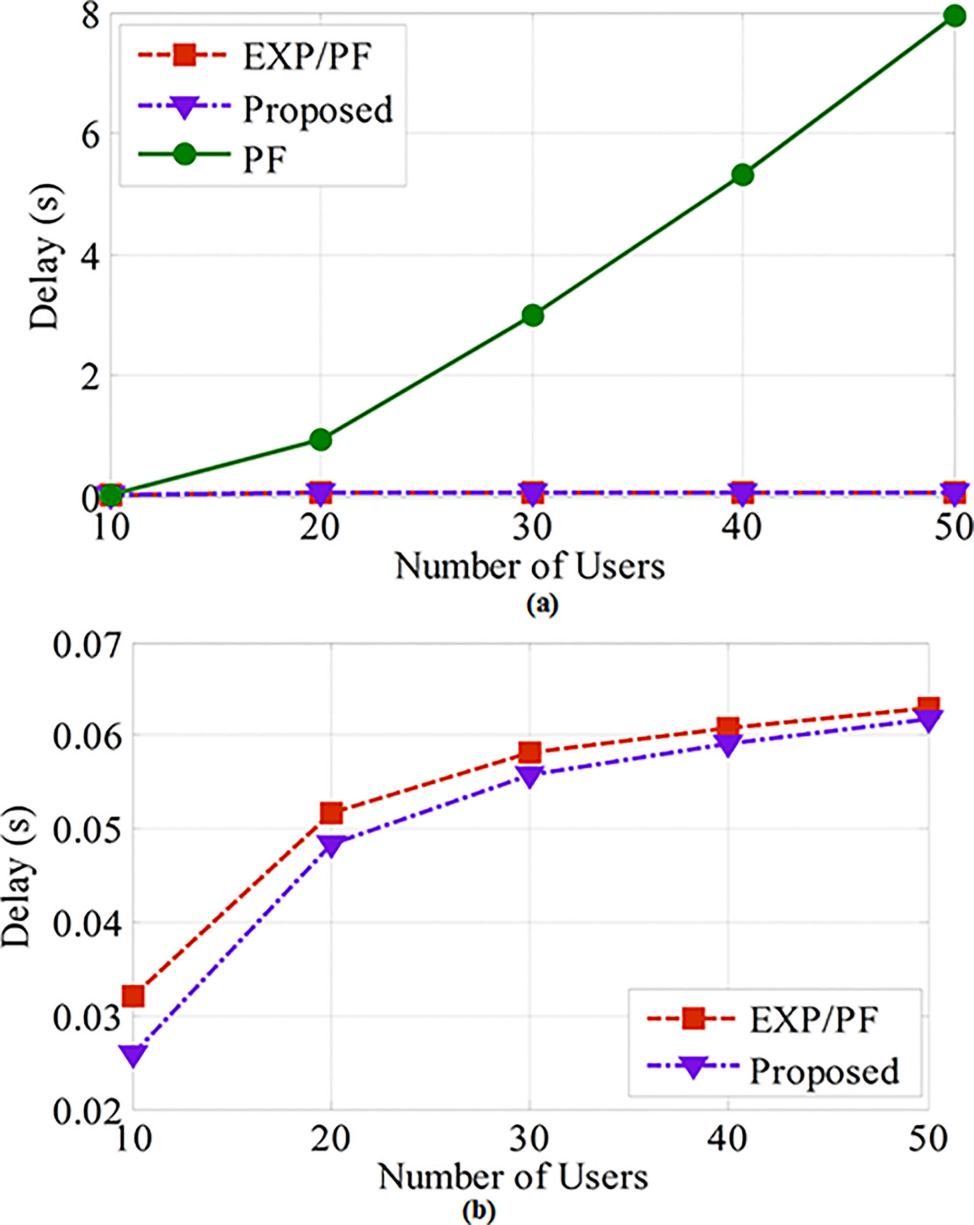
The delay for Class A and B applications. (A) Illustrates the delay of blood pressure, temperature, and heart rate applications. (B) Shows the delay of the proposed and EXP/PF approaches for blood pressure, temperature, and heart rate applications.

Fig 7 shows that the proposed approach is more robust in terms of the delay because the delay weighting factor does not allow the delay metric of the user’s packet to grow beyond the threshold level.

The EXP/PF approach shows nearly the same ability as the proposed approach to serve Class A users within the delay boundary because the priority of the application grows exponentially with respect to the growth of the packets’ delay. Compared with the other approaches, the PF approach shows the worst performance. This phenomenon can be attributed to the fact that PF is not concerned with the delay, which explains the sharp growth in the delay value as the number of sensors gradually increases. This is a clear indication of the PF’s inability to serve the application within its category of users.

**Fig 8:**
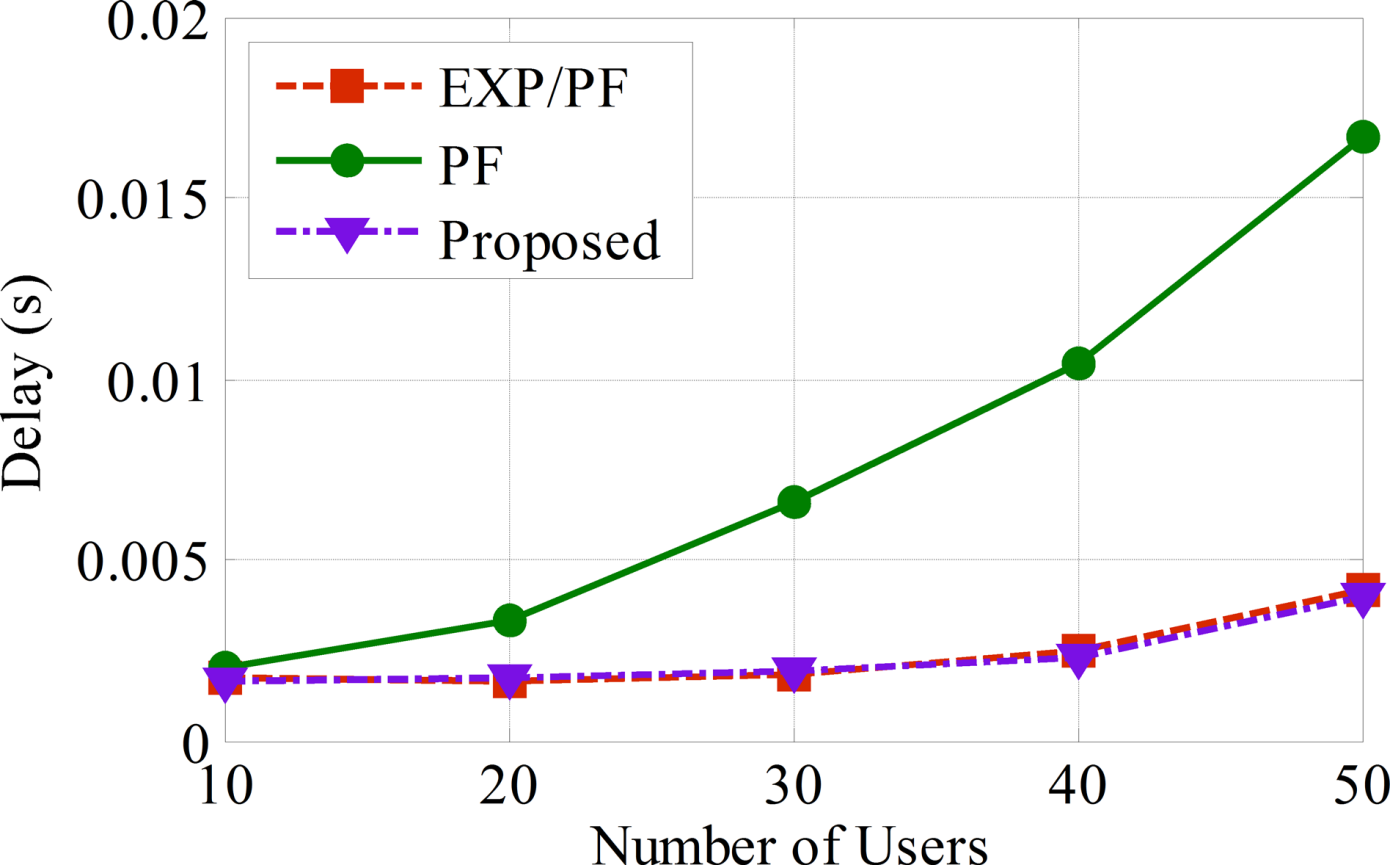
The delay for ECG application.

From the analyses of the class B delay metric, it can be concluded that the proposed and EXP/PF approaches show identical performance in terms of the delay, and this is because both approaches consider the delay metric. In contrast, the PF approach shows a substantial increase as the number of sensors increases (Fig 8).

**Fig 9:**
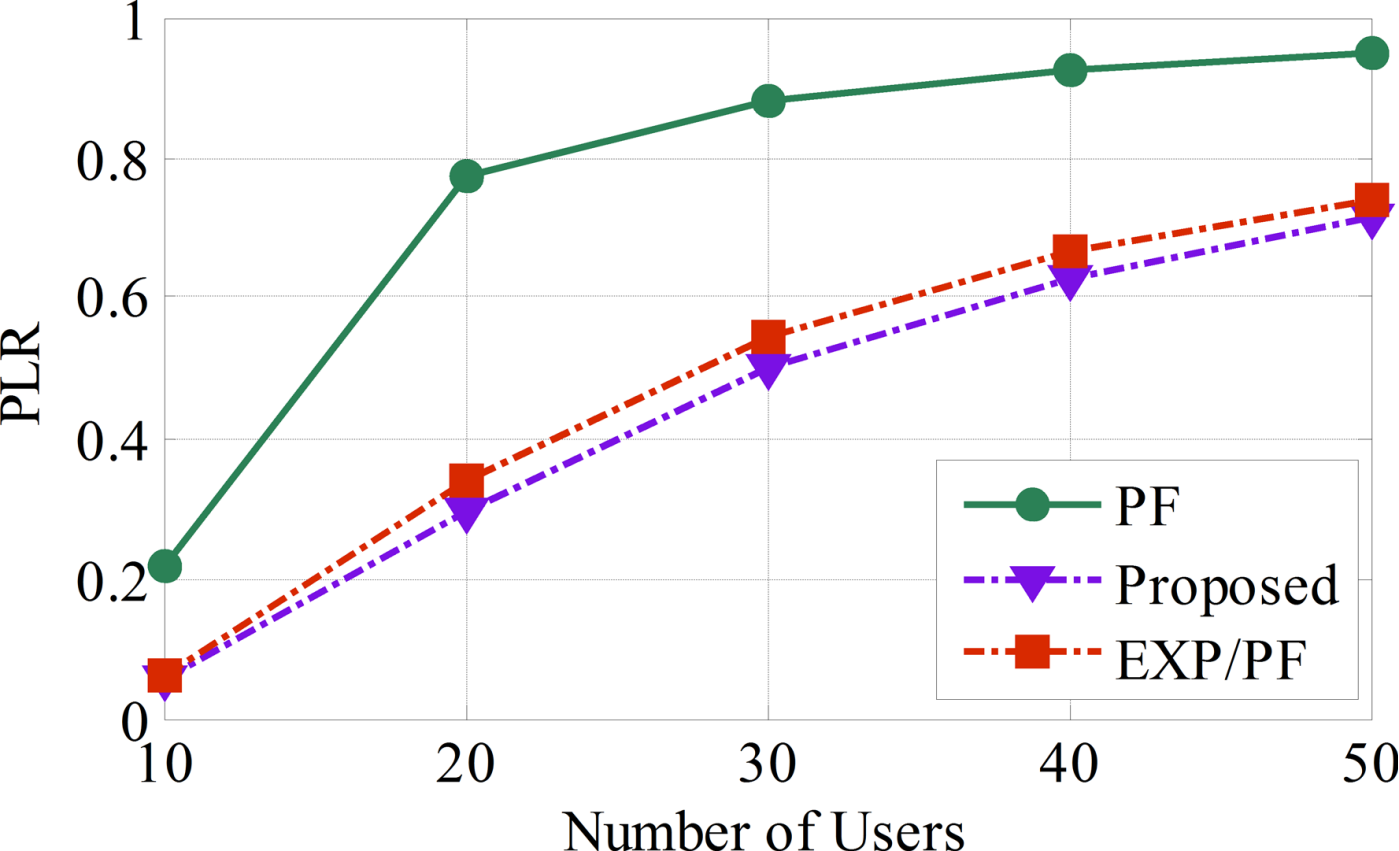
The PLR for blood pressure, temperature, and heart rate applications.

In Fig 9, the proposed algorithm shows the lowest Packet Loss Ratio (PLR), which proves that the proposed algorithm has the ability to maintain a good quality of service for Class A sensors. This ratio also shows relatively low growth for the proposed algorithm compared with other algorithms. At the 50th sensor, the PLR of the proposed algorithm shows an improved performance of up to 2% and 20% compared with EXP/PF and PF, respectively.

From Fig 9, we can conclude that the proposed approach shows the lowest PLR compared with the PF and EXP/Rule approaches. This is because the proposed approach is designed to serve sensors within the delay boundaries. The PF approach shows a significant increase in the number of dropped packets as the number of sensors asking for the service increases.

**Fig 10:**
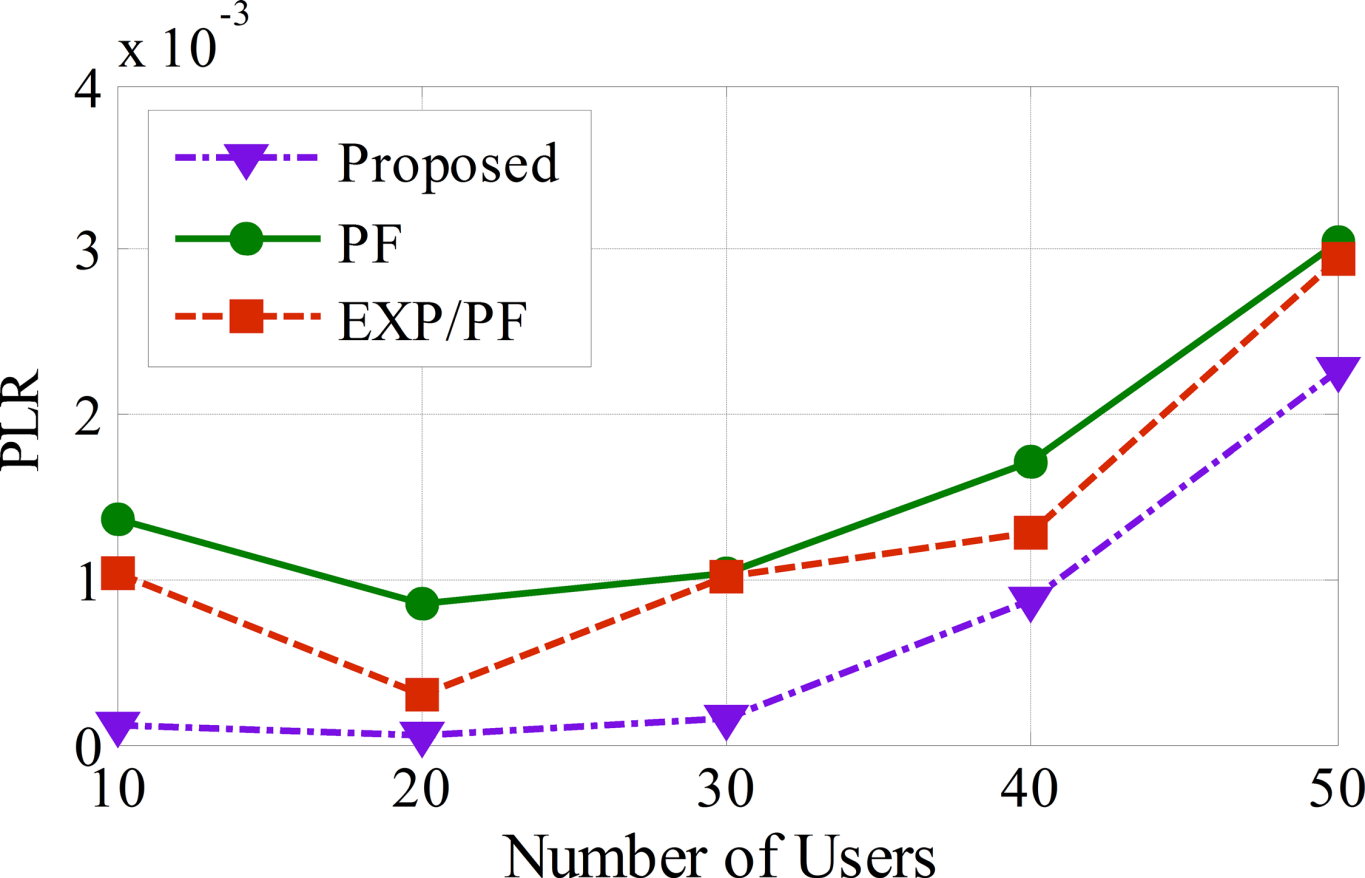
The PLR for ECG application.

As shown in Fig 10 for the ECG application, the proposed approach shows the lowest PLR compared with the other two approaches, which is as low as 25% for 50 sensors. This also shows the high efficiency in the resource management of the proposed approach, indicating that the proposed approach strictly considers the PLR factor in its scheduling decision metric by ensuring that sensors with PLR values closer to the drop threshold are served first. In contrast, the PF approach shows the worst performance because it does not consider the PLR factor.

## Limitations and Future Work

The main objective of the proposed algorithm is to distribute the resource blocks in an efficient and heuristic manner to meet the specific need of every individual application. Several limitations of the LTE-Femto cell network have been identified in terms of resource distribution, which is why in some cases, the scheduler assigns all or most of the RBs to only higher priority applications without considering lower priority applications. This results in a congestion case because the lower priority applications will have to wait longer or, in the worst case, suffer denial of services.

In the future, the scheduling approach will be extended and modified to work efficiently with future technology, such as LTE-A Femto cell networks. This can be achieved by adjusting the simulation environments and operating frequencies along with the introduction of a unique handover approach that will enable the mobile station to function smoothly in any heterogeneous network (LTE-Femto, LTE-A Femto, and WiMAX Femto networks). By doing so, we can guarantee that once the scheduler runs out of resources, the high priority applications will have the options to choose whether to join other practical base stations, i.e., the mobile station has the choice to be served by any available technology that it prefers. We are considering fusing the system together with cloud computing systems to guarantee high robustness in terms of resilience, privacy protection, and hardware failure issues. Based on the model proposed in Ref. [35], we propose a modeling framework based on a Heterogeneous Mean-Field (HMF) approximation to separate two concurrent diseases that work together with each other and to extend the work to cover homogeneous and heterogeneous networks in a similar manner to Refs. [35] and [36].

## Conclusion

This paper focused on the application of the IoT in modern healthcare systems for remote monitoring. As a first step, an android application was developed to act as a communication interface between the sensors and LTE-Femto cell networks. At the second level, a new scheduling approach was proposed based on the dynamic scheduling technique. This new approach had the ability to serve sensors in different categories based on the priority for each class as well as to guarantee the delay boundary to serve sensors within the applications demands, in terms of throughput, delay, and packet loss rate.

For up to 50 sensors, the simulation results showed that the proposed scheduling technique achieved a higher system throughput performance of up to 28% for the ECG application. Moreover, the proposed approach showed the best performance in terms of the PLR ratio, i.e., 5% and 12% for Class A and Class B, respectively.

## Supporting Information

S1 Fig General Structure of the Android Applications Process. (TIF)

S2 Fig PF and EXP/PF for Class A applications. (TIF)

S3 Fig PF and EXP/PF for Classes B application. (TIF)

S1 Text Data Collection and Android Applications. (DOCX)
