# Supplementary figures and images for: Enabling Remote Health-Caring Utilizing IoT Concept over LTE-Femtocell Networks
Source: PLoS One. 2016 May 6;11(5):e0155077. doi: 10.1371/journal.pone.0155077 (PMC4859479; doi:10.1371/journal.pone.0155077)

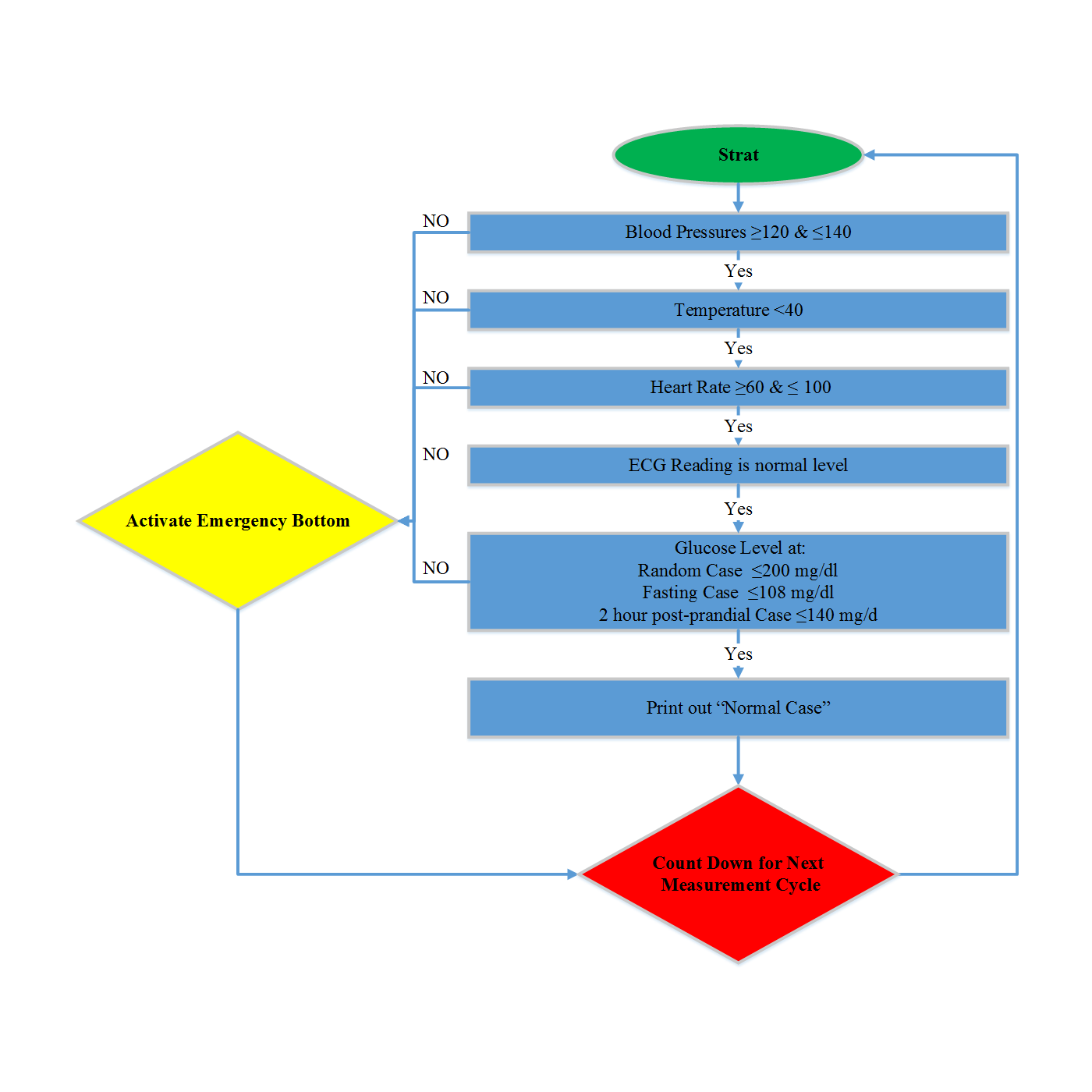

Supplement: S1 Fig — (TIF) [file pone.0155077.s001.tif]

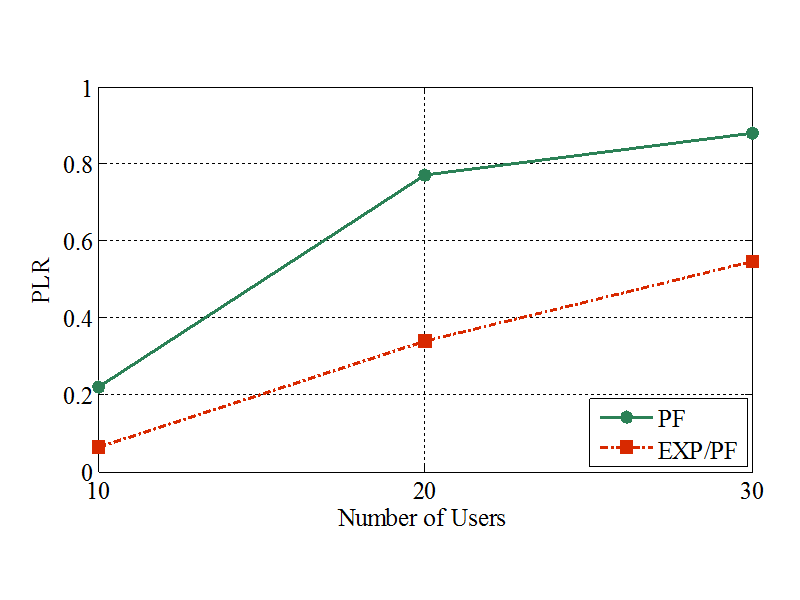

Supplement: S2 Fig — (TIF) [file pone.0155077.s002.tif]

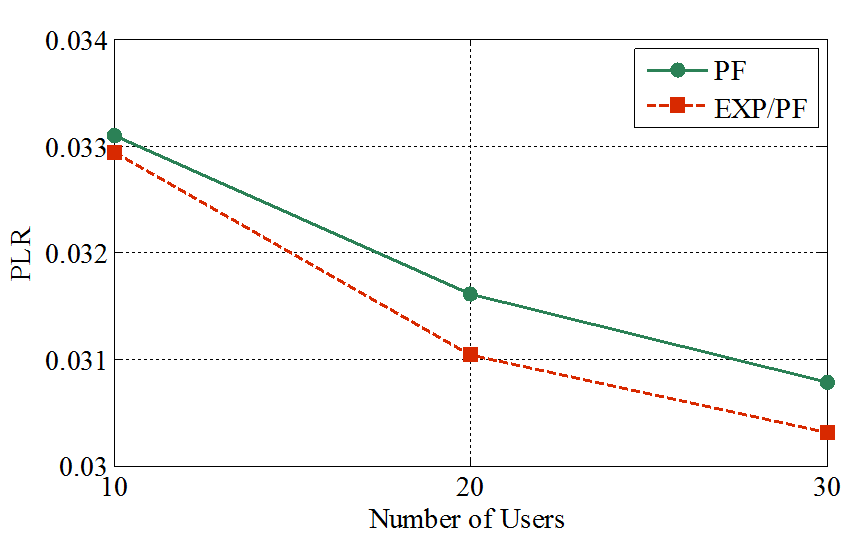

Supplement: S3 Fig — (TIF) [file pone.0155077.s003.tif]
