# Supplementary material for: Enabling Remote Health-Caring Utilizing IoT Concept over LTE-Femtocell Networks
Source: PLoS One. 2016 May 6;11(5):e0155077. doi: 10.1371/journal.pone.0155077 (PMC4859479; doi:10.1371/journal.pone.0155077)
Supplement: S1 Text — (DOCX) [file pone.0155077.s004.docx]

**Supporting Information**

**Enabling Remote Health-Caring Utilizing IoT Concept over LTE-Femtocell Networks**

M. N. Hindia^1,*^, T. A. Rahman^1^, H. Ojukwu^2^, E. B. Hanafi^2^, A. Fattouh^3^

^1^Wireless Communication Centre, Universiti Teknologi Malaysia, Skudai 81310, Malaysia

^2^Department of Electrical Engineering, Faculty of Engineering, University of Malaya, Kuala Lumpur, Malaysia

^3^Department of Computer Sciences, Faculty of Computing and Information Technology, King Abdulaziz University, Jeddah, Saudi Arabia

^*^Corresponding author

E-mail: nourhindia@hotmail.com

**Contents**

**1.** **Acronyms and Abbreviations** ……………………………………………….………2

**2.** **Data Collection and Android Applications** …………………………………..…….2

2.1 **Mobile Application Module** ………………………………………..…..……3

**3.** **Data Transmission over a Femto-LTE Network** ………………………….…...…..4

3.1 **The Existing Scheduling Algorithm** ………………………………………..5

3.2 **An Illustrated Example**.....………………………………………………...…6

**4.** **The Proposed Algorithm** …………………………………………………….……...8

1. **Acronyms and Abbreviations**

The list of acronyms and abbreviations used in the main text and this Supporting Online Material:

| IoT: | Internet of Things | |
| --- | --- | --- |
| AP: | Access Point | |
| LTE: | Long Term Evolution | |
| WiMAX: | Worldwide Interoperability for Microwave Access | |
| WLAN: | Wireless Local Area Network | |
| WPAN: | Wireless Personal Area Network | |
| WSN: | Wireless Sensor Network | |
| MAM: | Mobile Application Module | |
| PF: | Proportional Fairness | |
| EXP/PF: | Exponential Proportional Fairness | |
| EXP-Rule: | Exponential Rule | |
| PLR: | Packet Loss Ration | |
| TTI: | Transmission Time Interval | |
| Class A sensors: | Real-Time (For High priority applications) | Patients with Hypertension and other heart related disorders |
| Class B sensors: | Non-Real-Time ( For Mid priority applications) | Patients with Diabetic and related cases |
| Class C sensors: | Non-Real-Time (For Mid priority applications) | Patients with other types of sicknesses |

1. **Data Collection and Android Applications**

With the increasing danger of Hypertension, Diabetes and other related medical conditions, the need to develop a system that is capable of monitoring patients states of health any-time and any-where becomes crucial, as stated by Jie Wang, et al. in [1],

*“Hypertension, for instance, is a very critical public health issue worldwide because of its high prevalence and attendant increase in the risk of heart diseases [2]. Essential hypertension (EH) is a predisposing risk factor for stroke, myocardial infarction, congestive heart failure, arterial aneurysm, and the leading cause of chronic renal failure [3-5]. Approximately 90% to 95% of hypertension, affecting>1 billion adults worldwide, is the essential hypertension subtype [6]. About 5 to 10% of hypertension, relative to the case of primary hypertension, refers to the secondary hypertension, renal, endocrine and nervous system disease.”*

Henceforth, we conceive the idea of proposing a system of systems which has the ability to monitor remotely the patients, i.e., hypertensive patients, diabetic patients and patients with other sicknesses. After consulting with some specialist doctors, we arranged the sensors’ data priorities as will be described in the following section.

- 1. **Mobile Application Module**

The mobile application is responsible for collecting the data from different sensors attached to a patient’s body to decide whether the patient is in an emergency or non-emergency situation. The data is collected periodically from the body sensors and wirelessly forwarded to the android application where it is classified to determine the patient’s status. If all the readings at that moment is normal, the application will state “Normal Case” and no action will be taken. On the other hand, if the sensors’ measurements show any drop in value behind the Normal Case, then the emergency bottom will be triggered as shown in S1 Fig.

**S1 Fig.** **General Structure of the Android Applications Process.**

1. **Data Transmission over a Femto-LTE Network**

In most cases, after the data collection phase, there is a need to transmit the collected data over a robust, mature and well-tested network for remote administration by remote specialists. In this case, we chose the LTE network as a transmission based network. We also selected the Femto-cell network type since it is the most suitable network for hospital scenarios with a coverage area of up to 25 m (maximum on AP per room), and is well-known for low power consumption (suitable for sensors).

As a next step, the data transmission phase is proposed. After carful searching and surveying for the most used existing scheduling approaches [7], we have selected the PF and EXP-PF scheduling approaches [8-11] as a scheduling strategies to convey the patients’ measurements data over the Femto-LTE network to remote monitoring stations.

- 1. **The Existing Scheduling Algorithm**

The most popular scheduling schemes have been studied and compared with the ones applied in our own job. In Refs. [12], PF is studied. The scheduling metric for PF is based primarily on prioritizing users with high spectral efficiency. PF is known for its ability to guarantee high degree of fairness among users (1). The fairness is guaranteed by using the past average throughput as a weighting factor to ensure that all users are fairly served. The past average throughput gives the scheduler the idea about how much resources were required for the optimal performance of a practical user (sensor) in terms of throughput at previous TTIs. However the delay metric does not consider the scheduling decision metric.

(1)

Where is the scheduling metric, $\beta$is the constant factor which is inversely related to the window size ().

The EXP/PF is proposed as a solution to the existing issues for the real time applications [8]. The main idea behind this scheduling scheme is to distinguish users based on their packets types. For the real time users, it is based on combining the benefits of the exponential function which guarantees the delay boundaries of real time applications, while maximizing the system throughput. The robustness of this algorithm is as a result of its ability to take into account the exponential end to end delay of a user’s packets, thus, the scheduling metric grows exponentially along with the delay metric (2).

While the non-real time users are served as PF but with controlled degree of freedom (3), this degree increases or decreases based on the number of waiting packets of the real time applications ($w\left(t\right)$) at base station. The main drawback of this algorithm is based on its positive probability of service drops from the non-real time applications as shifting to the real time ones.

(2)

(3)

where is the head of line for sensor *i* at time *t*, $N.RT$ is the number of real time-application sensors.

- 1. **An Illustrated Example**

Form the S2 and S3 Figs, with a case study of up to 30 sensors and in a coverage area of up to 25 m, we can gain a meaningful insight into the behaviors of both EXP/PF and PF as the number of sensors increases. For EXP/PF in S2 Fig, as the number of Class A sensors increases, the PLR also increases, which means that the rest of the sensors such as sensor for Classes B and C applications are also allocated with some resource blocks the same time. From S3 Fig, we can see that as the number of Classes B and C sensors increases, the PLR decreases. In other word, we can say that, the delay metric of Classes B and C applications come close to their delay boundaries at 20 users, and that makes the scheduling decision metric to grow rapidly. From this point, we can understand the main drawbacks of the various existing scheduling approaches in terms of data transmission based on packets types. We have proposed a new approach of sending the sensors’ data over the LTE-Femto cell networks within the delay boundaries and with a guarantee to serve users based on their optimal resource preferences.


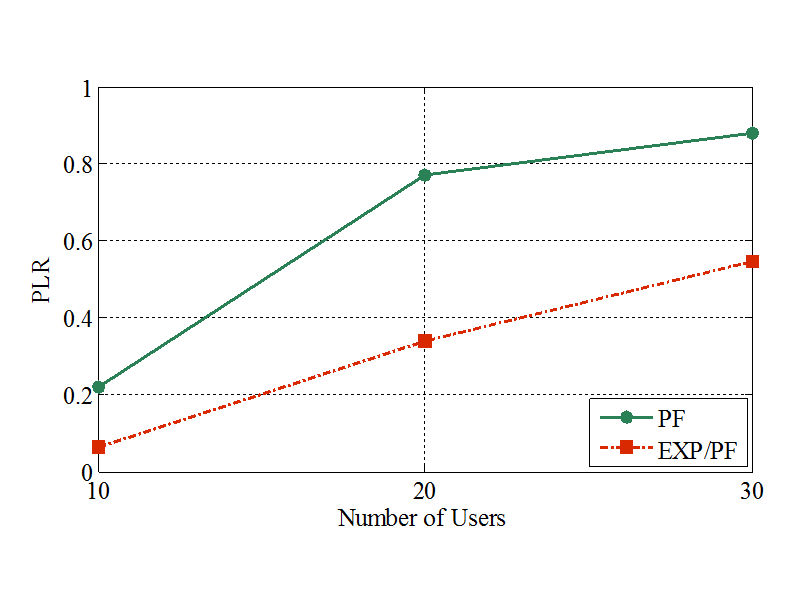


**S2 Fig. PF and EXP/PF for Class A applications.**


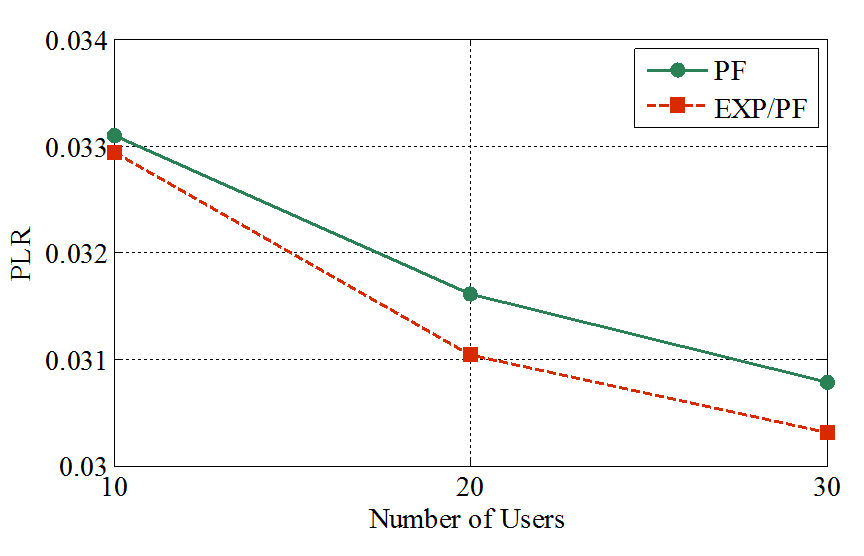


**S3 Fig. PF and EXP/PF for Classes B application.**

1. **The Proposed Algorithm**

The strength of this proposed model is based on the following factors; throughput factor, which takes into consideration the spontaneous data rates and the past average throughput. And the dynamic weighting factor, which is directly related and controlled by the patient’s status. These factors allow the system to change the scheduling priority from one TTI to another, depending on the patient’s / remote-doctor’s demands. For instance, we need to change from hypertension to the case of a diabetic patient, the dynamic application weight () will prioritize the Class B applications. And since the delay demands of this application will be weighted based on its new demands, the weighting factor of sensor *i* () will be re-calculated as the ratio between the head of the line packet delay of sensor *i* and the delay threshold of practical Class B will add up to another level to prioritize that application as well. It is worth mentioning that, the set of weight among the three classes is equal to 1.

**References**

1. Wang J, Xiong X, Liu W. Yoga for essential hypertension: a systematic review. PloS one. 2013;8(10):e76357.

2. Slama M, Susic D, Frohlich ED. Prevention of hypertension. Current Opinion in Cardiology. 2002;17(5):531-6.

1. Pierdomenico SD, Di Nicola M, Esposito AL, Di Mascio R, Ballone E, Lapenna D, et al. Prognostic value of different indices of blood pressure variability in hypertensive patients. American Journal of Hypertension. 2009;22(8):842-7.
2. Kumar S, Qu S, Kassotis JT. Effect of Omega-3 Polyunsaturated Fatty Acid Supplementation in Patients with Atrial Fibrillation. JAFIB: Journal of Atrial Fibrillation. 2012;5(2).
3. Chobanian AV, Bakris GL, Black HR, Cushman WC, Green LA, Izzo JL, et al. Seventh report of the joint national committee on prevention, detection, evaluation, and treatment of high blood pressure. Hypertension. 2003;42(6):1206-52.

7. Capozzi F, Piro G, Grieco LA, Boggia G, Camarda P. Downlink packet scheduling in LTE cellular networks: Key design issues and a survey. Communications Surveys & Tutorials, IEEE. 2013;15(2):678-700.

8. Basukala R, Mohd Ramli HA, Sandrasegaran K, editors. Performance analysis of EXP/PF and M-LWDF in downlink 3GPP LTE system. Asian Himalayas Regional International Conference on INTERNET; 2009.

9. Kim K, Koo I, Sung S, Kim K, editors. Multiple QoS support using M-LWDF in OFDMA adaptive resource allocation. Local and Metropolitan Area Networks, , The 13th IEEE Workshop on LANMAN; 2004.

10. Hindia MN, Reza AW, Noordin KA, Chayon MHR. A Novel LTE Scheduling Algorithm for Green Technology in Smart Grid. PloS one. 2015;10(4):e0121901.

11. Hindia MN, Reza AW, Noordin KA. A novel scheduling algorithm based on game theory and multicriteria decision making in LTE network. International Journal of Distributed Sensor Networks. 2015:14.

12. Lee S-B, Pefkianakis I, Meyerson A, Xu S, Lu S, editors. Proportional fair frequency-domain packet scheduling for 3GPP LTE uplink. INFOCOM 2009, IEEE.
